# The association between coffee consumption and periodontitis: a cross-sectional study of a northern German population

**DOI:** 10.1007/s00784-021-04208-9

**Published:** 2021-10-07

**Authors:** Julia Struppek, Carolin Walther, Kübra Kaymaz, Birgit-Christiane Zyriax, Jan-Per Wenzel, Juliana Senftinger, Julius Nikorowitsch, Guido Heydecke, Udo Seedorf, Thomas Beikler, Katrin Borof, Carola Mayer, Ghazal Aarabi

**Affiliations:** 1 Department of Prosthetic Dentistry, Center for Dental and Oral Medicine, University Medical Center Hamburg-Eppendorf, Hamburg, Germany; 2 Department of Periodontics, Preventive and Restorative Dentistry, University Medical Centre Hamburg-Eppendorf, 20246 Hamburg, Germany; 3 Midwifery Science – Health Service Research and Prevention, Institute for Health Services Research in Dermatology and Nursing (IVDP), University Medical Center Hamburg-Eppendorf (UKE), Hamburg, Germany; 4 Department of General and Interventional Cardiology, University Heart and Vascular Center, Hamburg, Germany; 5 Epidemiological Study Center, University Medical Center Hamburg-Eppendorf, Hamburg, Germany; 6 Department of Neurology, University Medical Center Hamburg-Eppendorf, Hamburg, Germany

**Keywords:** Coffee, Periodontitis, Oral health, Gingival recession, Gingival pocket, Cross-sectional studies, Confounding factors, Epidemiologic

## Abstract

**Background:**

Positive and negative influences on oral health are attributed to coffee consumption. The aim of the current study is to evaluate the association between coffee consumption and periodontitis in the general population of Hamburg.

**Methods:**

A total of 6,209 participants from the Hamburg City Health Study were included in this cross-sectional study. Information on coffee consumption was collected using a food frequency questionnaire. Periodontal examination included assessment of dental care ability via Plaque Index, measurement of pocket depth, gingival recession, and bleeding on probing. Classification was based on the criteria of Eke and Page. Ordinal logistic regression models were performed unadjusted and adjusted for confounding variables.

**Results:**

Periodontal cohort consists of 6,209 participants, presenting either none/mild (*n* = 1,453, 39.6% men, 2.4% strong coffee drinkers), moderate (*n* = 3,580, 49.3% men, 3.3% strong coffee drinkers), or severe (*n* = 1,176, 60.9% men, 5.0% strong coffee drinkers) periodontitis. There was a significant association between strong coffee consumption (≥ 7or more cups/day) and periodontitis (OR: 1.51; CI: 1.07, 2.12; *p* > 0.001), compared with low coffee consumption. Conversely, moderate coffee consumption was not associated with periodontitis, compared with low coffee consumption.

**Conclusion:**

and clinical relevance.

In this cross-sectional study of a northern German population, strong coffee consumption was significantly associated with periodontitis. Influence of changes in coffee consumption on periodontal disease etiology/progression should be investigated in future prospective study designs, in order to identify strong coffee consumption as a potential risk factor of periodontitis.

**Supplementary Information:**

The online version contains supplementary material available at 10.1007/s00784-021-04208-9.

## Introduction

Oral diseases affect approximately half of the global population, with severe periodontitis being one of the most prevalent non-communicable diseases [1–3]. Periodontitis is a chronic inflammatory disease of the tooth supporting tissues, characterized by deep periodontal pockets, bleeding gingiva, and alveolar bone loss. Dysbiosis of the oral microbiome [4] is suggested to induce a disproportionate host inflammatory and immune reaction (e.g., interleukin–6, tumor necrosis factor alpha, C‐reactive protein [5, 6]), resulting in periodontal damage in susceptible individuals. Additionally, genetic predisposition [7], epigenetics [8], lifestyle [9, 10], nutrition [11, 12], and presence of chronic inflammation [13] are independent risk factors for disease progression. Coffee is one of the most consumed beverages worldwide [14]. A total of 395 mL/day (~ 2 cups per day) of coffee are consumed in Germany [15]. Although coffee consumption is so common, the literature is still reporting both protective [16] and destructive [17] mechanisms to preserve/degrade periodontal tissue. Caffeine has antioxidant and anti-inflammatory effects by reducing reactive oxygen species and serum pro-inflammatory cytokines, respectively [16]. Chlorogenic acid from coffee demonstrated a direct inhibitory effect on *Porphyromonas gingivalis*, a major pathogen key-player of periodontitis [16] and can indirectly affect the bioavailability of other nutrients and thereby modulate periodontal disease prevalence [18]. A few studies suggest an association between coffee intake and periodontitis [19–23]. The US Department of Veterans Affairs Dental Study included 1,231 participants and reported no harmful but rather a beneficial effect of coffee on periodontal health [22]. A Korean population-based study reported higher tooth loss in participants with daily coffee intake, compared with those only drinking coffee less than once a month [19].

Therefore, the aim of the current study is to evaluate the potential association between coffee consumption and periodontitis using state-of-the-art, comprehensive phenotyping derived from the Hamburg City Health Study (HCHS).

## Material and methods

### Study participants and design

The Hamburg City Health Study (HCHS) is an ongoing prospective, population-based cohort study at the University Medical Center Hamburg-Eppendorf which commenced 2016 with the receipt of the ethical approval (PV5131). The study has been registered at ClinicalTrials.gov (NCT039934957). HCHS aims to investigate several important risk and prognostic factors in major chronic diseases [24]. The HCHS is in accordance with the current revision of the Declaration of Helsinki [25], the ethical principles described by Good Clinical Practice (GCP) and by Good Epidemiological Practice (GEP). Participants between 45 and 74 years of age from the general population of Hamburg were recruited. Written informed consent was obtained from all participants. Inclusion criteria included a completed periodontal examination and documentation on coffee consumption data. Exclusion criteria included individuals requiring endocarditis prophylaxis.

### Determination of coffee consumption parameters

Coffee consumption was assessed using a previously validated food frequency questionnaire (FFQ) [26] that included cups of coffee consumed per day for the last 12 months, type of coffee (caffeinated or decaffeinated), and additives (no additives, sweetener, milk, evaporated milk, sugar, or honey). Multiple answers were possible for type of coffee and additives. Frequency of coffee consumption was classified as low (0–2 cups/day), moderate (3–6 cups/day), and strong (≥ 7or more cups/day).

### Determination of periodontal parameters

Oral and periodontal examination was carried out by trained and calibrated study nurses. The decayed, missing, and filled teeth (DMFT) index was recorded and periodontal examination was carried out with a PCP-12 probe except for third molars. Probing depths (PD) and gingival recessions (GR) were recorded in 6 sites (mesio-oral, oral, disto-oral, mesio-buccal, buccal, disto-buccal) for each tooth in millimeters. Clinical attachment loss (CAL) was calculated (CAL = PD + GR). Bleeding on probing (BOP) and Plaque Index (PI) was recorded accordingly [27]. The diagnosis and the severity of periodontitis (none/mild, moderate and severe) was classified according to the criteria of Eke and Page [28]:

### Additional variables

The additional variables, such age, sex, education (based on international standard classification of education [29]), and smoking (non-smoker, former smoker [quit smoking at least 6 month ago], current smoker), were assessed via self-reported questionnaire. In the study center, the following variables were measured: body mass index (BMI in kg/m^2^), diabetes mellitus (taking medication of the A10 group (ATC-Code), fasting glucose > 126 mg/dl, non-fasting glucose > 200 mg/dl, positive self-disclosure), coronary artery disease (CAD was defined as suffering from one or more of the following conditions: status post myocardial infarction, percutaneous coronary intervention (PCI) or history of coronary bypass surgery), hypertension (was defined as a systolic blood pressure ≥ 140 mmHg, a diastolic blood pressure ≥ 90 mmHg, or the use of one or more of the following antihypertensive drugs: ACE inhibitors, angiotensin II receptor blockers, beta blockers, calcium channel blockers, renin inhibitors, or loop diuretics), and laboratory parameters (serum high-sensitive IL-6 and high-sensitive CRP).

### Statistical analyses

Descriptive analyses were performed for all variables stratified by periodontitis severity. Continuous variables were presented as median and interquartile range [median (IQR)] and categorical variables were presented as absolute numbers and percentages [*n* (%)]. Ordinal logistic regression models were used to test hypotheses and to determine the association between periodontitis (dependent variable) and coffee consumption (independent variable; low coffee consumption served as reference group). Models were stepwise adjusted (model 1: unadjusted; model 2: adjusted for confounders: age, sex, smoking, diabetes, and hypertension). Results of the associations are reported as odds ratios (OR) with 95% confidence intervals (95% CI) and *p*-values. All statistical analyses were performed in Page R software version 4.1.0 (R Project for Statistical Computing) with *p*-values < 0.05 interpreted as statistical significance.

## Results

### Descriptive statistics

Overall cohort consists of 10,000 participants with 48.9% being men with a median age of 63 years. Of the participants, 63.3% of them were low (0–2 cups/day), 33.3% moderate (3–6 cups/day), and 3.5% strong (≥ 7 cups/day) coffee drinkers. Periodontal cohort consists of 6,209 participants, presenting either none/mild (*n* = 1,453, 39.6% men, 2.4% strong coffee drinkers), moderate (*n* = 3,580, 49.3% men, 3.3% strong coffee drinkers), or severe (*n* = 1,176, 60.9% men, 5.0% strong coffee drinkers) periodontitis. The baseline characteristics of the study participants stratified by periodontitis severity are presented in Table 1 (and categorized for coffee consumption as Table S1). Participants with severe periodontitis were more often men (60.9%) with median age of 66 years, 4.1% exhibit a lower education, and 25.1% were currently smoking. Furthermore, 11.3% were diabetic and 72.5% suffered from hypertension. Five percent of participants with severe periodontitis drunk ≥ 7 cups of coffee per day.

**Table 1:** Baseline characteristics of the study population classified by the degree of periodontitis

**Characteristics**		**Periodontitis**		
	Overall cohort	None/mild PA	Moderate PA	Severe PA
Number of participants	10,000	1,453	3,580	1,176
**Median [IQR] or n (%)**				
**Socio-demographic characteristics**				
Female sex	5,108 (51.1)	878 (60.4)	1,814 (50.7)	460 (39.1)
Age	63 [55, 70]	59 [52, 66]	63 [55, 69]	66 [59, 71]
Education				
Low	313 (3.4)	43 (3.1)	151 (4.4)	55 (5.0)
Medium	4,801 (52.4)	675 (48.5)	1,711 (50.0)	605 (54.5)
High	4,052 (44.2)	675 (48.5)	1,559 (45.6)	450 (40.5)
**Cardiovascular risk factors**				
Smoking				
Current	1,978 (19.9)	235 (16.2)	608 (17.1)	293 (25.1)
Former	4,406 (44.3)	625 (43.2)	1,581 (44.4)	550 (47.0)
Never	3,565 (35.8)	588 (40.6)	1,369 (38.5)	326 (27.9)
BMI	26.13 [23.53, 29.21]	25.56 [23.01, 28.67]	26.02 [23.55, 29.01]	26.45 [24.11, 29.65]
Diabetes mellitus	794 (8.6)	85 (6.2)	242 (7.4)	122 (11.3)
Coronary artery disease	498 (5.1)	46 (3.2)	140 (4.0)	62 (5.3)
Hypertension	6,301 (66.1)	768 (54.8)	2,266 (66.3)	810 (72.5)
**Laboratory parameters**				
Hs CRP	0.12 [0.06, 0.26]	0.10 [0.06, 0.23]	0.11 [0.06, 0.25]	0.13 [0.07, 0.30]
Hs IL6	1.64 [1.18, 2.39]	1.47 [1.03, 2.08]	1.57 [1.16, 2.23]	1.80 [1.34, 2.69]
**Coffee parameters**				
**Coffee consumption caffeinated**				
Low	5,699 (63.3)	843 (63.7)	2,026 (62.1)	666 (63.4)
Moderate	2,999 (33.3)	449 (33.9)	1,127 (34.6)	331 (31.5)
Strong	311 (3.5)	32 (2.4)	107 (3.3)	53 (5.0)
With milk	5,428 (63.4)	843 (67.4)	1,973 (63.6)	617 (61.5)
With evaporated milk	769 (9.0)	99 (7.9)	287 (9.3)	90 (9.0)
With sugar	1,126 (13.2)	160 (12.8)	377 (12.2)	130 (13.0)
With honey	77 (0.9)	10 (0.8)	20 (0.6)	11 (1.1)
With sweetener	419 (4.9)	62 (5.0)	135 (4.4)	43 (4.3)
Black	2,843 (33.2)	377 (30.1)	1,025 (33.0)	358 (35.7)
**Coffee consumption decaffeinated**				
Low	8,787 (98.5)	1,300 (99.0)	3,188 (98.7)	1,010 (97.5)
Moderate	125 (1.4)	13 (1.0)	40 (1.2)	25 (2.4)
Strong	13 (0.1)	0	3 (0.1)	1 (0.1)
With milk decaf	1,231 (60.4)	191 (63.0)	454 (61.6)	118 (53.2)
With evaporated milk decaf	158 (7.7)	21 (6.9)	59 (8.0)	23 (10.4)
With sugar decaf	208 (10.2)	37 (12.2)	55 (7.5)	20 (9.5)
With honey decaf	22 (1.1)	3 (1)	8 (1.1)	3 (1.4)
With sweetener decaf	100 (4.9)	18 (5.9)	35 (4.7)	10 (4.5)
Black decaf	658 (32.3)	88 (29)	236 (32)	84 (37.8)
**Dental parameters**				
DMFT Index	20 [16, 23]	17 [14, 21]	19 [16, 23]	21 [17, 24.25]
BOP	7.69 [1.92, 20.37]	2.08 [0, 7.14]	8.33 [2.17, 19.23]	21.05 [9.26, 41.67]
Plaque Index	8.7 [0, 29.17]	0 [0, 10.71]	8.7 [0, 27.78]	21.74 [5.77, 54.76]

Abbreviations: *BMI* body mass index; *BOP* bleeding on probing; *DMFT Index* Decayed, Missing, Filled, Teeth Index; *Hs CRP* high-sensitivity C-reactive protein; *Hs IL-6* high-sensitivity interleukin-6; *decaf* decaffeinated

### Association between coffee consumption and periodontitis

Ordinal logistic regression analyses revealed significant association between strong coffee consumption and periodontitis in the unadjusted (OR: 1.52; 95% CI: 1.10, 2.09; *p* > 0.001) and adjusted (age, sex, smoking, diabetes, and hypertension) model (OR: 1.51; CI: 1.07, 2.12; *p* > 0.001) in comparison with low coffee consumption (Table 2). Conversely, moderate (3–6 cups/day) coffee consumption was neither associated in the unadjusted nor in the fully adjusted model with periodontitis, compared with low coffee consumption.

**Table 2:** Ordinal logistic regression models: outcome periodontitis, exposure coffee consumption (*caffeinated*)

Parameter	OR	95% CI	*p*-value
**Model 1**			
Moderate coffee consumption	0.9538	0.8424, 1.0799	0.456
Strong coffee consumption	1.5202	1.1044, 2.0913	> 0.001
**Model 2**			
Moderate coffee consumption	1.0196	0.8908, 1.1669	0.7782
Strong coffee consumption	1.5071	1.0692, 2.1244	> 0.001
Age	1.0511	1.0426, 1.0598	< 0.001
Female sex	0.6798	0.5986, 0.7717	< 0.001
Current smoking	1.8983	1.5798, 2.2823	< 0.001
Former smoking	1.2579	1.0955, 1.4447	> 0.001
Diabetes mellitus	1.001	0.7845,1.2771	0.9936
Hypertension	1.3129	1.1452, 1.5055	0.7782

Model 1: unadjusted; model 2: adjusted for age, sex, smoking, diabetes, and hypertension. *CI* confidence interval, *OR* odds ratio

## Discussion

The current study included comprehensive phenotypical data from 6,209 participants with complete periodontal examination and documentation of coffee consumption in order to determine a potential association. Participants with severe periodontitis were more often men with median age of 66 years. After adjusting for potential confounders (age, sex, smoking, and diabetes mellitus), strong coffee consumption was significantly associated with periodontitis compared to participants with low coffee consumption. In contrast, moderate (3–6 cups/day) coffee consumption was not associated with periodontitis in comparison to low coffee consumption.

Few studies confirmed a negative association of coffee on oral health [19, 20, 30–32]. Longitudinal data from the Korean National Health and Nutrition Examination Survey (KNHANES) (n = 16,730) revealed higher consumption of coffee in men with periodontitis [20]. Highest coffee consumption (≥ 3 cups/day) in the KNHANES study overlaps with moderate coffee consumption (3–6 cups/day) defined for the current study. In contrast, the current study revealed no significant association between moderate coffee consumption and periodontitis when compared to low coffee consumption. But, the small fraction of ‘strong coffee consumers’ (> 7 cups/day) were significantly associated with periodontitis. The literature describes a potential mechanism: caffeine causes harmful effects on bone metabolism by directly influencing the signaling cascade that leads to cell death of osteoblasts [33] and/or indirectly by increased excretion [34] or decreasing absorption [35] of calcium.

Varying periodontal classification protocols challenge a meaningful comparison of our results to prior studies. For example, two cross-sectional studies observed a significant association between daily coffee consumption and tooth loss [19, 32], thus reporting solely the absolute endpoint of disease manifestation. In the current study, we used the gold standard to report periodontal diseases in epidemiological studies, classifying in no/mild, moderate, and severe periodontitis [36].

The significant association between strong coffee consumption and periodontitis remained significant after adjusting for age, sex, smoking, diabetes and hypertension. In line with other studies, high coffee consumption was significantly associated with a higher level of cigarette smoking, an important risk factor for periodontal disease [37, 38]. Smoking induces vasoconstriction and negatively influence fibroblast functions and induce collagen breakdown in periodontal tissues [39, 40]. Thus, we included smoking as a confounder variable and were still able to demonstrate association between coffee consumption and periodontitis. We also excluded the following covariables from regression analyses: education (proven evidence for no association with coffee consumption [41]) and Plaque Index (detected multicollinearity; variance inflation factors = 6.9).

Furthermore, the confounder “stress” could not be included in our regression model, though it’s proven association to periodontitis in clinical [42] as well as epidemiological studies [43]. The effect of stress on the body/periodontitis is mostly mediated by a complex hormonal response system in the hypothalamus–pituitary–adrenal axis (HPA). In brief: activation of the HPA results in increased levels of cortisol, which can (1) inhibit T-cell immune response and thereby amplify the outgrowth of pathogenic micro-organism [43], (2) act as oxidative damage mediator, which can promote periodontal disease progression [44], (3) influences bone metabolism via apoptosis of osteocytes [44].

### Limitations

The current findings showed an association between strong coffee consumption and periodontitis; however, a cross-sectional study design cannot address causality. Furthermore, findings derived from the northern German population cannot necessarily be generalized to other populations. Another limitation is the small sample size in participants drinking only decaffeinated coffee (strong consumption of decaffeinated coffee *n* = 16). Analyses for potential associations between decaffeinated coffee and periodontitis could not be performed.

## Conclusion

In this cross-sectional study, strong but not moderate coffee consumption was significantly associated with periodontitis, compared to participants with low coffee consumption. Influence of changes in coffee consumption on periodontal disease etiology and progression should be investigated in future prospective study designs, in order to identify strong coffee consumption as a risk factor of periodontitis and its progression.

## Supplementary Information

Below is the link to the electronic supplementary material.

Supplementary file1 (DOCX 19.2 KB)

## Declarations

### **Clinical Trial Registration**:

The study has been registered at ClinicalTrials.gov (NCT039934957).

The present study is reported according to the STROBE Guidelines [45]. All authors agree to be held accountable for all aspects of the present study.
