# Supplementary material for: The association between coffee consumption and periodontitis: a cross-sectional study of a northern German population
Source: Clin Oral Investig. 2021 Oct 7;26(3):2421–7. doi: 10.1007/s00784-021-04208-9 (PMC8898214; doi:10.1007/s00784-021-04208-9)
Supplement: Supplementary file 1 — Supplementary file1 (DOCX 19.2 KB) [file 784_2021_4208_MOESM1_ESM.docx]

**Supplementary Material**

**Supplementary Table 1: Baseline characteristics of the study population classified by coffee consumption**

| **Characteristics** |  | **Coffee consumption** | | |
| --- | --- | --- | --- | --- |
|  | Overall cohort | Low | Moderate | Strong |
| Number of participants | 10,000 | 5,699 | 2,999 | 311 |
| **Median [IQR] or n (%)** |  |  |  |  |
| **Socio-demographic characteristics** |  |  |  |  |
| Female sex | 5,108 (51.1) | 3,099 (54.4) | 1,404 (46.8) | 107 (34.4) |
| Age | 63 [55, 70] | 64.00 [57.00, 70.00] | 60.00 [53.00, 67.00] | 59.00 [54.00, 65.00] |
| Education |  |  |  |  |
| Low | 313 (3.4) | 254 (4.7) | 92 (3.2) | 15 (5.0) |
| Medium | 4,801 (52.4) | 2,784 (51.1) | 1,453 (50.3) | 157 (52.0) |
| High | 4,052 (44.2) | 2,406 (44.2) | 1,341 (46.5) | 130 (43.0) |
| **Cardiovascular risk factors** |  |  |  |  |
| Smoking |  |  |  |  |
| Current | 1,978 (19.9) | 827 (14.6) | 783 (26.2) | 121 (39.2) |
| Former | 4,406 (44.3) | 2,574 (45.4) | 1,292 (43.2) | 133 (43.0) |
| Never | 3,565 (35.8) | 2,266 (40.0) | 914 (30.6) | 55 (17.8) |
| BMI | 26.13 [23.53, 29.21] | 25.96 [23.41, 29.09] | 26.12 [23.63, 29.15] | 27.51 [24.91, 30.14] |
| Diabetes mellitus | 794 (8.6) | 478 (9.1) | 192 (7.0) | 24 (8.4) |
| Coronary artery disease | 498 (5.1) | 395 (9.4) | 160 (7.1) | 27 (11.4) |
| Hypertension | 6,301 (66.1) | 3,732 (68.3) | 1,732 (61.0) | 173 (60.1) |
| **Laboratory parameters** |  |  |  |  |
| Hs CRP | 0.12 [0.06, 0.26] | 0.12 [0.06, 0.26] | 0.11 [0.06, 0.26] | 0.12 [0.07, 0.25] |
| Hs IL6 | 1.64 [1.18, 2.39] | 1.61 [1.18, 2.31] | 1.55 [1.14, 2.20] | 1.83 [1.23, 2.49] |
| **Coffee parameter** |  |  |  |  |
| **Coffee consumption caffeinated** |  |  |  |  |
| With milk | 5,428 (63.4) | 3,453 (65.6) | 1,795 (60.1) | 180 (58.1) |
| With evaporated milk | 769 (9.0) | 490 (9.3) | 252 (8.4) | 27 (8.7) |
| With sugar | 1,126 (13.2) | 738 (14.0) | 355 (11.9) | 33 (10.6) |
| With honey | 77 (0.9) | 61 (1.2) | 16 (0.5) | 0 (0.0) |
| With sweetener | 419 (4.9) | 261 (5.0) | 142 (4.8) | 16 (5.2) |
| Black | 2,843 (33.2) | 1,567 (29.8) | 1,143 (38.3) | 133 (42.9) |
| **Coffee consumption decaffeinated** |  |  |  |  |
| Low | 8,787 (98.5) | 5,578 (99.3) | 2,891 (97.1) | 298 (96.1) |
| Moderate | 125 (1.4) | 33 (0.6) | 87 (2.9) | 3 (1.0) |
| Strong | 13 (0.1) | 4 (0.1) | 0 (0.0) | 9 (2.9) |
| With milk decaf | 1,231 (60.4) | 811 (62.2) | 381 (58.1) | 30 (47.6) |
| With evaporated milk decaf | 158 (7.7) | 106 (8.1) | 47 (7.2) | 5 (7.9) |
| With sugar decaf | 208 (10.2) | 141 (10.8) | 60 (9.1) | 4 (6.3) |
| With honey decaf | 22 (1.1) | 18 (1.4) | 4 (0.6) | 0 (0.0) |
| With sweetener decaf | 100 (4.9) | 67 (5.1) | 28 (4.3) | 4 (6.3) |
| Black decaf | 658 (32.3) | 387 (29.7) | 239 (36.4) | 25 (39.7) |
| **Dental parameter** |  |  |  |  |
| **Periodontitis severity** |  |  |  |  |
| None/Mild | 1,453 | 843 (23.8) | 449 (23.5) | 32 (16.7) |
| Moderate | 3,580 | 2,026 (57.3) | 1,127 (59.1) | 107 (55.7) |
| Severe | 1,176 | 666 (18.8) | 331 (17.4) | 53 (27.6) |
| DMFT Index | 20 [16, 23] | 19.00 [16.00, 23.00] | 19.00 [15.00, 23.00] | 20.00 [16.00, 24.00] |
| BOP | 7.69 [1.92, 20.37] | 7.89 [2.00, 20.37] | 7.14 [1.92, 18.52] | 9.62 [2.08, 25.00] |
| Plaque Index | 8.7 [0, 29.17] | 8.33 [0.00, 29.17] | 7.69 [0.00, 26.89] | 10.71 [0.00, 37.50] |

Abbreviations: BMI = body mass index, BOP = bleeding on probing, DMFT Index = Decayed, Missing, Filled, Teeth Index, Hs CRP = high-sensitivity C-reactive protein; Hs IL-6 = high-sensitivity interleukin-6; decaf = decaffeinated
